# Supplementary material for: Cardiolipin deficiency disrupts electron transport chain and drives steatohepatitis
Source: eLife. 2026 Sep 2;14:RP106976. doi: 10.7554/eLife.106976 (PMC13537762; doi:10.7554/eLife.106976)
Supplement: Supplementary file 4 [file elife-106976-fig1-data3.zip › Figure 1-source data 3.docx]

**Figure 1– source data 3**

Patient demographic information

|  | Healthy | MASH |
| --- | --- | --- |
| Age at time of collection | 50.3 ± 9.6 yrs | 62.2 ± 7.1 yrs |
| Sex | Male: 1 Female: 10 | Male: 9 Female: 8 |
| Alcohol use? | N/A | Yes: 0 No: 17 |
| Race | White: 9 African American: 1 Asian: 1 | White: 10 Unknown: 7 |
