# Supplementary material for: Cardiolipin deficiency disrupts electron transport chain and drives steatohepatitis
Source: eLife. 2026 Sep 2;14:RP106976. doi: 10.7554/eLife.106976 (PMC13537762; doi:10.7554/eLife.106976)

Figure 3H

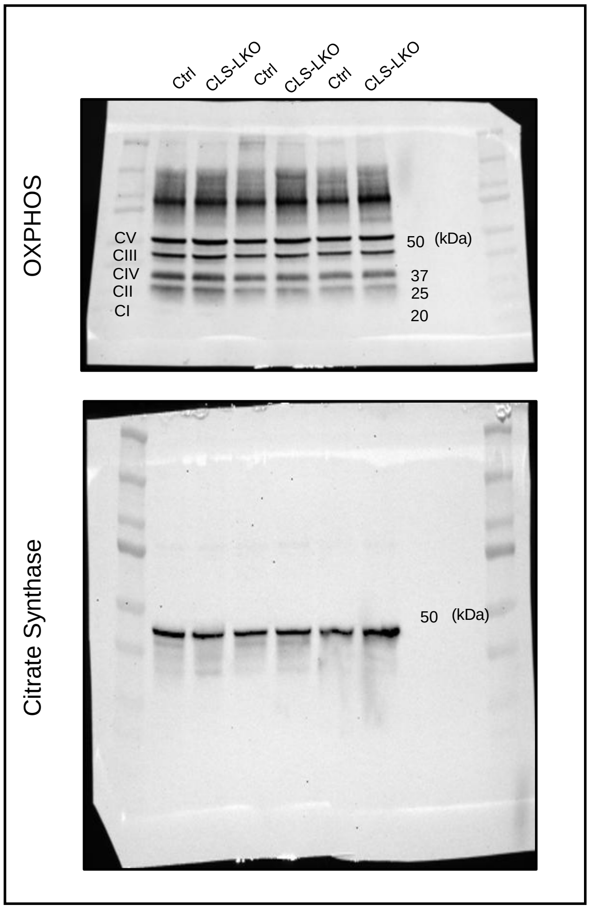

Figure 3M

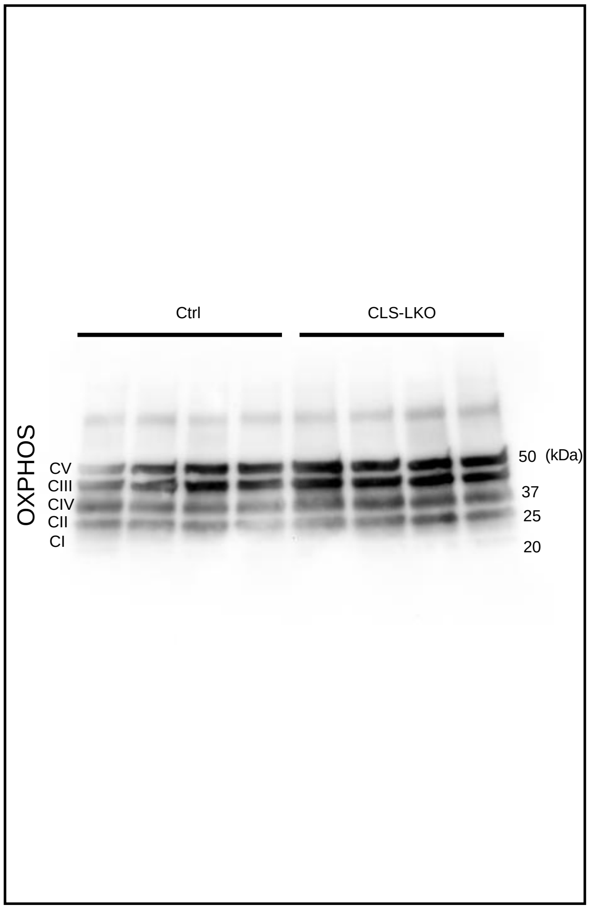

Supplement: Supplementary file 5 [file elife-106976-fig3-data1.zip › Figure 3-source data 1.pdf]
