# Supplementary material for: Cardiolipin deficiency disrupts electron transport chain and drives steatohepatitis
Source: eLife. 2026 Sep 2;14:RP106976. doi: 10.7554/eLife.106976 (PMC13537762; doi:10.7554/eLife.106976)

Figure 6A

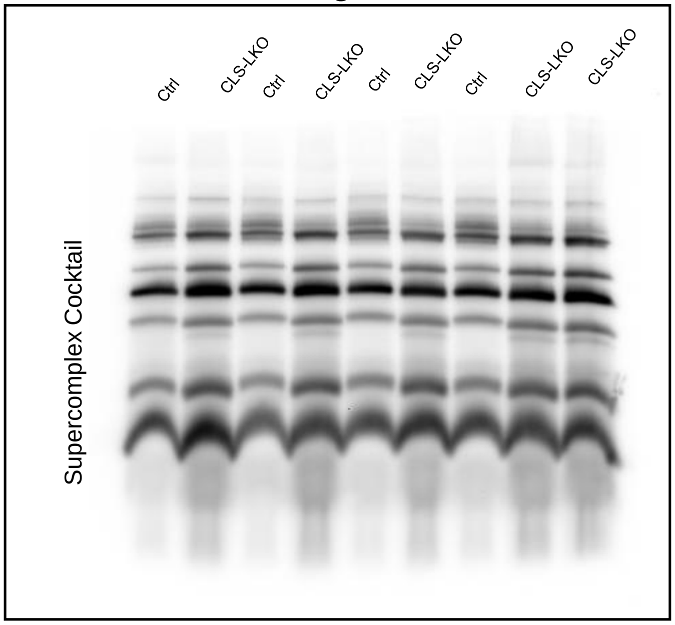

Figure 6B

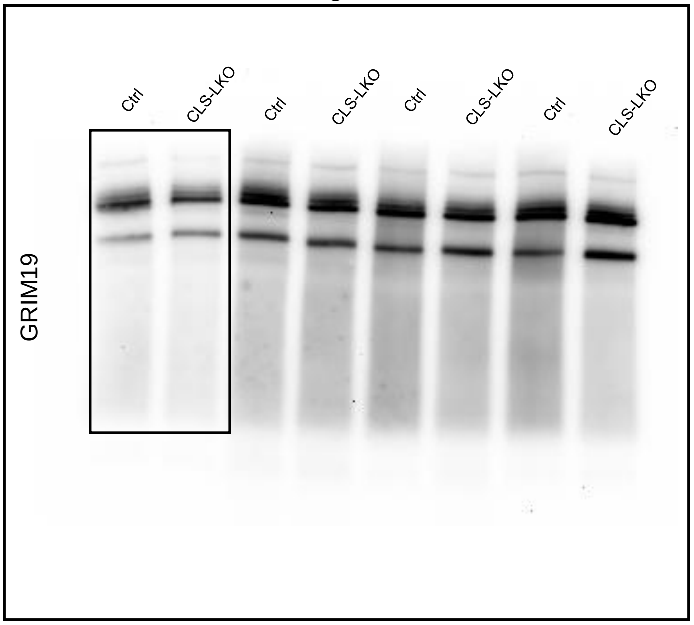

Figure 6D

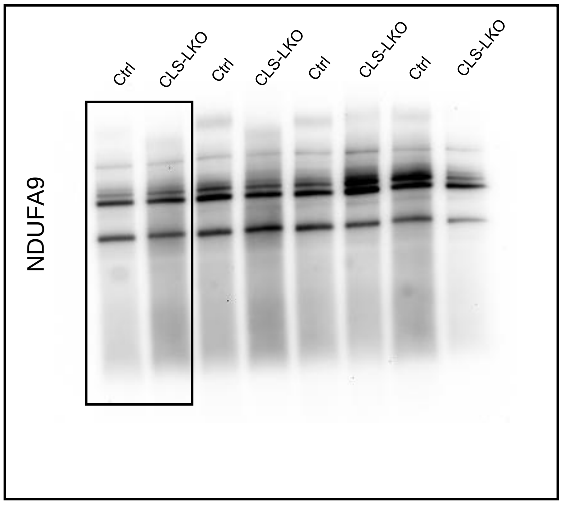

Figure 6F

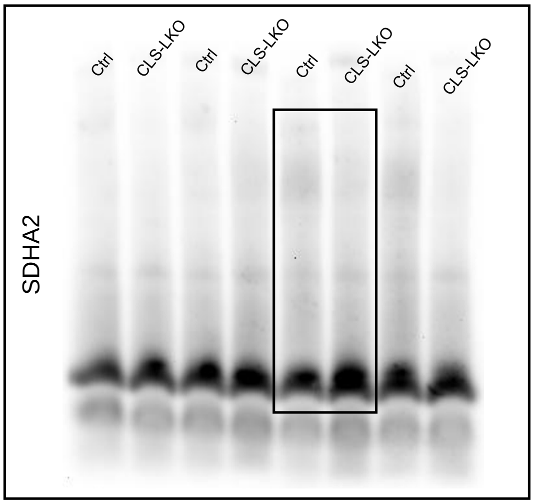

Figure 6H

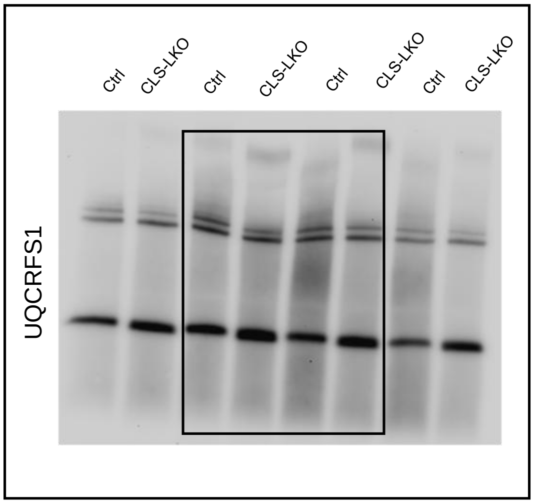

Figure 6J

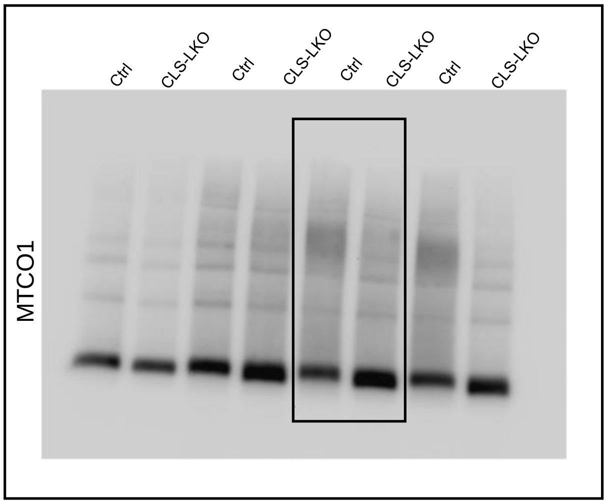

Figure 6L

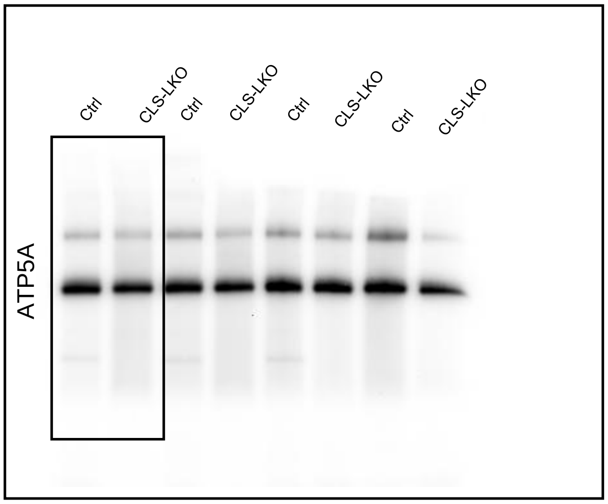

Supplement: Supplementary file 11 [file elife-106976-fig6-data1.zip › Figure 6-source data 1.pdf]
