# Supplementary material for: Cardiolipin deficiency disrupts electron transport chain and drives steatohepatitis
Source: eLife. 2026 Sep 2;14:RP106976. doi: 10.7554/eLife.106976 (PMC13537762; doi:10.7554/eLife.106976)
Supplement: Peer review history

# Editor report: eLife Assessment

**DOI:** 10.7554/eLife.106976.3.sa0

**Authors:** David Ron

**Affiliations:** University of Cambridge United Kingdom

**Keywords:** Convincing, Solid

This revised manuscript retreats from the original claim of establishing a causal link between cardiolipin deficiency and the progression from steatotic liver disease to steatohepatitis and instead advances a more limited mechanistic conclusion: that cardiolipin deficiency perturbs electron transport and promotes electron leak from the mitochondrial respiratory chain. The experimental evidence supporting this revised claim is now **solid**, and the potential for increased electron leak to contribute to liver pathophysiology is demonstrated. However, absent evidence that cardiolipin deficiency is causally upstream of disease progression, the overall significance of the work remains limited. While the study provides a **convincing** analysis of mitochondrial bioenergetics, the narrowing of its central claim diminishes its impact relative to that proposed in the original submission.

# Referee report: Joint Public Review

**DOI:** 10.7554/eLife.106976.3.sa1

Cardiolipin, is a key lipid constituent of mitochondrial membranes. Perturbation of its abundance is thus poised to affect broad aspects of mitochondrial function. Given the important role of mitochondria, it is not surprising that cardiolipin deficiency would have pervasive effects on cell physiology.

The original version of this paper advanced the idea that cardiolipin deficiency, and the attendant mitochondrial dysfunction, plays a causative role in the progression of fatty liver (a common feature in the human population) to a more pathogenic inflammatory state known as steatohepatitis. Given the prevalence of this form of liver disease in the human population this claim for discovery was deemed sufficiently interesting to merit peer review at eLife.

Peer review reaffirmed the importance of the claim but also revealed important limitations in the experimental support provided. Specifically, the lack of experimental interventions that uncouple the correlation between progression in a mouse model and changes in cardiolipin abundance to test the causal relationship. The review process also recognised the utility of other aspects of the paper, namely the evidence implicating cardiolipin deficiency in altered properties of the mitochondrial membrane, its contribution to an electron leak and the potential for these features to contribute to pathology.

The revised version of the manuscript now focuses on the importance of cardiolipin sufficiency to mitochondrial integrity and contains various improvements to the data supporting this aspect. At the same time the revised paper retreats from the most interesting claim of a causal role for cardiolipin deficiency in disease progression. We are left with a more convincing but less significant paper.

# Author comment: Author response

**DOI:** 10.7554/eLife.106976.3.sa2

**Authors:** Marisa J Brothwell, Guoshen Cao, John Alan Maschek, Annelise M Poss, Alek D Peterlin, Liping Wang, Talia B Baker, Justin L Shahtout, Piyarat Siripoksup, Quentinn Pearce, Jordan M Johnson, Fabian M Finger, Alexandre Prola, Sarah A Pellizzari, Gillian L Hale, Allison M Manuel, Shinya Watanabe, Edwin R Miranda, Kajsa E Affolter, Trevor S Tippetts, Linda S Nikolova, Ran Hee Choi, Stephen T Decker, Mallikarjun Patil, Leon J Catrow, Will L Holland, Sara M Nowinski, Daniel S Lark, Kelsey H Fisher-Wellman, Patrice N Mimche, Kimberley Evason, James E Cox, Scott A Summers, Zach Gerhart-Hines, Katsuhiko Funai

**Affiliations:** University of Utah Salt Lake City United States; University of Utah Salt Lake City United States; University of Utah Salt Lake City United States; University of Utah Salt Lake City United States; University of Utah Salt Lake City United States; University of Utah Salt Lake City United States; University of Utah Salt Lake City United States; University of Utah Salt Lake City United States; University of Utah Salt Lake City United States; University of Utah Salt Lake City United States; University of Utah Salt Lake City United States; University of Copenhagen Copenhagen Denmark; University of Grenoble Alpes Grenoble France; University of Utah Salt Lake City United States; University of Utah Salt Lake City United States; University of Utah Salt Lake City United States; University of Utah Salt Lake City United States; University of Utah Salt Lake City United States; University of Utah Salt Lake City United States; University of Utah Salt Lake City United States; University of Utah Salt Lake City United States; University of Utah Salt Lake City United States; University of Utah Salt Lake City United States; University of Utah Salt Lake City United States; University of Utah Salt Lake City United States; The University of Utah Salt Lake City United States; Van Andel Institute Grand Rapids United States; Colorado State University Fort Collins United States; Wake Forest University Winston-Salem United States; Indiana University Indianapolis United States; University of Utah Salt Lake City United States; University of Utah Salt Lake City United States; University of Utah Salt Lake City United States; University of Copenhagen, Denmark Copenhagen Denmark; University of Utah Salt Lake City United States

The following is the authors’ response to the original reviews.

As the reviewers noted, the evidence we provide is the strongest on the mechanistic link between hepatic cardiolipin deficiency and electron leak from the electron transport chain. This narrative is supported by our assessment of site-specific electron leak as well as reconstitution of exogenous cardiolipin in the small unilamellar vesicles deficient with CL. On the other hand, as pointed out by the Reviewer 2, the mechanistic link between cardiolipin to MASLD/MASH is less robust. At this moment, we have not experimentally demonstrated that the MASLD/MASH induced by CLS deletion can be rescued by replacement of mitochondrial CL in vivo. Taken together, our current narrative makes an incomplete loop between CL deficiency, electron leak, and MASLD/MASH. Nevertheless, as indicated by all the reviewers, this manuscript highlights a previously undescribed role that CL potentially plays in MASH pathology, particularly with the data that human MASH coincides with reduction in liver mitochondrial CL. We focused this revision primarily on additional descriptive experiments in CLS-LKO mice that were requested by the reviewers. Even though it is not a component of the current manuscript, we have recently successfully developed mice with hepatocyte-specific CLS overexpressing mice and began performing experiments to test causality of CL deficiency to MASLD/MASH which we hope to complete in a few years. We are hopeful that the MASLD/MASH research community will still find evidence on CL contained in this manuscript plausible, and that it provides critical information to our understanding of mechanisms for MASH pathogenesis.

**Public Reviews:**

**Reviewer #1 (Public review):**

Summary:

The manuscript by Brothwell and colleagues describes a central role for hepatic cardiolipin deficiency in MASH. The authors identify cardiolipin as a mediator of two long-standing problems in the field: how dysregulated lipid metabolism relates to altered mitochondrial metabolism during MASLD, and what the innate changes are in the steatotic liver that cause the increased respiration. The authors identified reduced liver cardiolipin in humans with MASH and in a variety of mouse models with MASH. When they knocked out hepatic cardiolipin synthesis, mice developed steatosis and inflammation. These mice also recapitulated the elevated hepatic oxidative metabolism and oxidative stress found in obese humans with MASLD. Some of the in vivo functional data related to glucose homeostasis and substrate metabolism could be stronger, and interpretation of the in vitro flux data needs some clarification, but in both cases, the data are not essential to the main conclusions of the manuscript. Overall, the study offers compelling evidence that cardiolipin is reduced in MASLD and that impaired cardiolipin synthesis is sufficient to recapitulate many features of MASLD.

We thank the reviewer 1 for the positive feedback emphasizing novel and important findings in our manuscript.

Strengths:

The main strengths of the study are:

(1) The identification of reduced cardiolipin levels in the liver of humans with MASLD and in a variety of mouse models of MASLD.

(2) The finding that loss of cardiolipin synthesis recapitulates steatosis and inflammation in MASH.

(3) The finding that loss of cardiolipin increases mitochondrial respiration, ROS production, and fat oxidation (in a separate hepatocyte cell line), again recapitulates several previous studies in obese humans with MASLD.

(4) Evidence, though less definitive, that cardiolipin deficiency promotes electron leak by disrupting respiratory supercomplexes and preventing CoQ reduction.

Weaknesses:

(1) Figure 3A-D tries to make the point that liver CLS KO causes defects in substrate handling in vivo, based on glucose and pyruvate tolerance tests. The KO mice have a blunted response to a glucose tolerance test, but the pyruvate tolerance test showed very little (almost no) effect on glucose levels in either WT or LKO mice. The small blunting of the response in the LKO is impossible to interpret (if it's real), since the ability to clear glucose is also increased, and no tracers were used. It might be useful to monitor pyruvate and lactate levels during the experiment. However, this reviewer doesn't think the data is essential to prove the authors' main points.

Thank you for pointing this out. We have now revised our manuscript to correctly reflect our findings on GTT and PTT. In our initial submission, we failed to clearly articulate that CLS deletion appeared to increase systemic glucose handling, which is the opposite of what one might expect in liver with steatosis. We agree that additional experiments would be helpful to better understand the systemic substrate handling in the CLS-LKO mice. As the reviewer indicates, we decided to focus this particular manuscript on intracellular and mitochondrial metabolism because of cardiolipin’s known localization to mitochondria, and the central role that this organelle plays in the pathogenesis of MASLD.

(2) After presenting convincing evidence that respiration is elevated in isolated mitochondria from CLS KO liver, the authors follow up the findings by investigating whether 13C-palmitate and 13C-glucose oxidation are altered by CLS knockdown in murine Hepa1-6 cells (Figure 4).

A few comments are worth mentioning about Figure 4:

(a) It is not clear why the authors chose to use a hepatoma cell line rather than primary hepatocytes from LKO mice. The latter would be more convincing, since there could be important differences in metabolism between hepatoma cells and hepatocytes (e.g., preference for fatty acids vs glucose). Nevertheless, I think the approach is sufficient to test the general effect of loss of CLS on substrate metabolism.

We appreciate the sentiment and agree that primary hepatocytes would have been a better model. We simply have not had prior expertise to culture primary hepatocytes and do not have the system working. We completely agree that it’s important to discuss the limitation of hepa1-6 cells as a hepatoma cells and now discuss this in our manuscript.

(b) The authors use the M+2 enrichments of TCA cycle intermediates to infer rates of oxidation of [U-13C] palmitate or [U-13C] glucose. It is important to note that this kind of data reports fractional carbon sources (i.e., substrate preference) rather than rates of oxidation. For example, data from the 13C-palmitate experiment indicates that the CLS KD cells increase the fractional contribution from 13C palmitate (compared to glucose, for example) to the TCA cycle, but the actual rate of palmitate oxidation is not implicit in the data. However, it is reasonable to suggest that, in combination with the increased rates of O2 consumption observed in isolated mitochondria, this data supports increased fat oxidation.

We agree with the reviewer that the nuances are important: that M+2 enrichments from [U-13C] palmitate or [U-13C] glucose reflects the fractional contributions of labeled substrates to the TCA cycle rather than oxidation. We have now revised the text to clarify that the data represent carbon incorporation patterns.

(c) I have some concern that the [U-13C] glucose experiment is more complicated to interpret than the description implies. I'm not sure what happens in this cell line, but in the liver, most labeling from pyruvate (i.e., originating from glucose in this case) enters the TCA cycle via pyruvate carboxylase, with smaller amounts entering via PDH (depending on the nutritional state). Since one could expect pyruvate carboxylase to contribute M+3 labeled TCA cycle intermediates initially, and M+2 on the first turn of the cycle, it's hard to conclude what the data indicates about glucose oxidation. The authors could generalize the conclusion by framing the TCA cycle enrichment data as the contribution of glucose carbons and noting in Figure 4A that pyruvate carbons can enter the TCA cycle via PDH or pyruvate carboxylase, without attempting to assign their relative contributions. There are better ways to do it, but it's a small nuance here since the authors aren't making a critical point about the pathways.

This expert comment is much appreciated. We have revised the text to more broadly describe glucose carbon entry into TCA cycle through PDH and PC. We also revised the schematic to reflect this notion.

**Reviewer #2 (Public review):**

In this study, the authors show that alterations in the lipid composition of the inner mitochondrial membrane, particularly changes in cardiolipin (CL) content, lead to defects in electron transport, supercomplex formation, and oxidative stress. Using liver-specific CLS knockout mice, which are characterized by dysfunctional capacity for cardiolipin synthesis, the authors highlight an underappreciated role for CL in MASH pathology. Overall, this is an interesting study highlighting the importance of functional/physiological electron transport (and in this context, electron leakage) in MASH pathophysiology. Despite that, this manuscript has several weaknesses that require attention.

We thank the reviewer 2 for the constructive criticisms and identifying areas of weakness were additional data or explanations can improve the manuscript.

(1) For all LKO studies, it is stated that the decrease in hepatic CL is causal for the observed phenotype. However, it is evident that many other lipids are impacted by CLS KO, including a marked increase in hepatic PG. In this respect, the authors show no evidence that the observed metabolic phenotype is indeed due to the reduction in CL and not to other accompanying changes.

Thanks for this comment. We agree that because deletion of CLS promotes changes in mitochondrial lipids other than CL, we cannot conclusively attribute phenotypes we observed to CL and not to other lipids such as PG. In our experience, rescuing mitochondrial phospholipids by exogenous supplementation is problematic as they most certainly are not exclusively destined to the tissue of interest, nor to the organelle of interest, and often metabolized to produce other lipids, etc, making it difficult to interpret the data. We now have mice that conditionally overexpress CLS, which could be used to address this question, but the study is in its early phase and are outside the scope of the current study.

The one experiment we performed is the ex vivo CL supplementation by SUV fusion to mitochondria, which has an ability to rescue electron leak. While they do not demonstrate the role of CL in all phenotypes found in the CLS-LKO mice, we think that bioenergetic phenotype associated with CLS deletion is therefore likely due to the reduction in CL. We now provide these additional discussions in lines.

(2) In the results, the authors highlight that 'MASLD has been shown to alter the total cellular lipidome in liver.' Given that this study focused on CL, it would be useful to include specific studies that pointed to changes in hepatic CL content in MASLD/MASH/fibrosis.

We now provide citations for these studies (PMID: 30042157, PMID: 34257827).

(3) The initial human mitochondrial lipidomics studies show a reduction in mitochondrial CL and PG content. What was the content/expression of CL synthase and PGP synthase in these samples? If this cannot be assessed, is there any association of CLS or PGPS expression and MASLD/fibrosis (etc) in publicly available databases (e.g, GEP liver) that may explain the reduction in mitochondrial PG and CL content?

Thanks for this suggestion. Quantification of mitochondrial lipidome require a good amount of tissue, and we do not have sufficient biomaterials left to quantify gene expression. Upon our survey of publicly available database (including GepLiver), we did not find that human MASLD was associated with an increase in CLS or other enzymes of CL biosynthesis compared to healthy controls.

(4) The validation of MASH in patients (Figure 1B) is not convincing (ie., no quantification/scoring provided). NAS /fibrosis scoring (according to Kleiner) would help to define if all patients have indeed MASH, and what subset has fibrosis. Could the reduction in CL/PG content be (also) associated with fibrosis? In addition, Masson's Trichrome should be added to Figure 1B.

The diagnosis was based on obvious bridging fibrosis and/or regenerative nodules on H&E staining (see additional zoomed-out images in Figure 1 – figure supplement 1). Due to the severity of these cases, formal NAS scoring was not applied. We do not have the Trichrome staining available but all MASH samples had fibrosis. Thus, it is possible that reduced CL/PG is related to fibrosis. We now added more descriptions on this point.

(5) In human lipidomics, the authors suggest that reductions are observed in tetralinoleoyl CL (Figure 1C). However, Figure 1C only shows the combined FA acyl chain length + unsaturation, therefore not allowing for FA-specific ID (unless such data are available from the LC/MS analysis).

Thanks for pointing this out. Per lipidomic nomenclature guideline we assign combined FA acyl chain length + unsaturation when MS2 is not performed. We have validated that our 72:8 peak corresponds to TLCL, but we do not perform MS2 on every lipid species for every sample. We now clarify this point in our manuscripts.

(6) Figures 1 J/K/I. It is obvious that the background in all murine immunoblotting analysis has been altered. The authors should provide unaltered images for these immunoblots.

We apologizes with the confusion. In Figure 1J/K/L/M, each panel actually represents two western blots (not one, similar to Figure 3H). The above represents a western blot with OXPHOS antibody cocktail (CV, CIII, CIV, CII, and CI), while the bottom represents the second western blot with citrate synthase (CS). Thus, we had not manipulated parts of the western blot to look different. To clarify, we now place an outline in each of the western blot to clearly demarcate individual blots to avoid confusion (new Figure 1J-M).

(7) For Figure 1, it is unclear what is meant by 'we performed all mitochondrial lipidomic analyses by quantifying lipids per mg of mitochondrial proteins'. Was the murine lipidomics carried out on fractionated mitochondria or whole liver? If whole liver, then how were the data corrected, particularly given that PG is not a mitochondria-specific lipid?

The data are all from lipidomic analyses performed in isolated mitochondria.

(8) While total CL content seems indeed decreased across the different mouse models, this is mostly due to 1-2 CL species showing a pronounced reduction, with the remainder being unaltered. This should at least be acknowledged in the results. This is similarly the case in the LKO livers.

Thanks for pointing this out. We now provide additional clarification in the text.

(9) Figure 2. A secondary biochemical analysis of changes in lipid content should be provided, e.g., total triglyceride content, particularly given that the histology analysis does not show any major changes in hepatic lipid droplets/steatosis. In addition, the Masson's Trichrome staining shows almost no collagen deposition.

We now provide a quantification of triglycerides in Figure 2J.

(10) Figure 3. 'CLS deletion modestly reduced glucose handling' should be reworded. The LKO mice show improved glucose tolerance (despite the MASH phenotype), which is not evident from the above wording.

We modified our text accordingly.

(11) Looking at the mechanism behind the increase in hepatic steatosis, the authors state that lipid accumulation can occur due to increased lipogenesis, or dysfunctional VLDL secretion or beta oxidation, and subsequently assessed the relevant proteins/pathways. What about fatty acid uptake, which is also one of the four major pathways impacted in MASLD? This should be included in this assessment in Figure 3.

Thank you for this comment. We now provide data for genes involved in fatty acid uptake, which was not reduced with CLS deletion (Figure 3E).

(12) For Figure 5A, it is simply stated 'CLS deletion promotes liver fibrosis in standard chow-fed condition', and it is unclear what is highlighted within the selected EM images and what the arrows refer to. The authors should clarify this within the text.

We have modified the text accordingly.

**Reviewer #3 (Public review):**

Summary:

Mitochondrial oxphos causes lipid accumulation, leading to MASH, although the mechanism has been poorly understood. In this study, Funai and colleagues identify that reductions in cardiolipin in the mitochondria cause disruptions in the electron transport chain. Knockout of cardiolipin synthase was sufficient to drive MASH phenotypes, increase respiratory capacity, and cause electron leak at complexes II and III. It is well established that loss of cardiolipin increases ROS. Studies to date have been performed on whole tissue lysates, but to rule out which changes in mitochondrial lipids are driven by changes in mitochondrial number versus lipid synthesis/turnover, the authors uniquely purified mitochondria from human and mouse livers in MASH and NASH models for this study. This study provides critical information to the field that will inevitably help us better understand the mechanisms underlying MASH and NASH onset. The evidence provided is both convincing and compelling. With further suggested revision experiments, this study has the potential to change our understanding of MASH and NASH pathogenesis.

We would like to thank the reviewer 3 for the highly-encouraging feedback.

Strengths:

The authors use a unique approach of lipidomics on purified mitochondria. They also analyze many distinct MASH models and provide a unique resource for the field of comprehensive lipidomics analysis of the different ways in which MASH can be induced. The use of human tissue elevates the impact/significance of the findings.

Weaknesses:

The data on the super complexes was the least compelling, and frankly, I do not think the authors needed those data to make a compelling argument! The authors should shift their focus more to the compelling electron leak data they have collected. If possible, it would also strengthen the work to include cardiolipin rescues on more of the experiments. Finally, expanding their explanations of the model systems would be very helpful for the readership.

Thank you for this comment. We have now revised our argument to highlight the electron leak data and less emphasis on the supercomplexes.

**Reviewer #4 (Public review):**

Summary:

Here, the authors wish to shed light on factors that contribute to the development of liver disease in what used to be called 'the metabolic syndrome'. This is a human-health problem of considerable significance, and the insights they provide, namely the implication of a defect in mitochondrial cardiolipin (CL) content to the progression from metabolic dysfunction associated steatotic liver disease to steatohepatitis, are plausible.

We would like to thank the reviewer 4 in an encouraging feedback.

Strengths:

The experimental evidence proffered is derived from the observation of lower levels of (CL) in mitochondria from the liver of patients undergoing liver transplant or resection due to endstage steatohepatitis compared with mitochondria derived from livers of patients with other conditions. This correlation is buttressed by observations made in mice with liver-selective compromise in CL synthesis and which suggest a pathological environment associated with mitochondrial dysfunction and enhanced oxidative stress, features deemed to play a role in the progression from steatotic liver disease to steatohepatitis.

The paper is well written, and the findings are well explained and superficially convincing.

Weaknesses:

It is unclear how much can be learned from compromising a key enzyme that produces a key mitochondrial lipid in a busy metabolic organ like the liver - isn't the discovery of a mitochondrial defect in such a context rather trivial? And how reliably can these findings be related to the human observations? Most importantly, the chain of causality implied by the title is unproven: the key question of whether or not (somehow) preventing the drop in cardiolipin content affects the course of steatohepatitis remains unanswered.

We agree with the reviewer that the current manuscript does not directly provide evidence that reduction in CL causes MASLD in humans, which as the reviewer describes, must be tested by rescuing CL content in the context of MASLD. We have now obtained mice with conditional overexpressor and have begun the experiments, but findings from these mice are beyond the scope of the current study. We have modified our title to “Cardiolipin deficiency disrupts electron transport chain AND drives steatohepatitis” to reduce the implication for causality.

**Recommendations for the authors:**

**Reviewer #1 (Recommendations for the authors):**

The manuscript states that loss of mitochondrial respiration is expected in MASLD. Forexample, line 187 "MASLD is known to be associated with reduced mitochondrial oxidative capacity". A more accurate statement is that "MASH" is known to be associated with reduced mitochondrial oxidative capacity and increased ROS production in humans. As you correctly cite later for an ex vivo human mitochondrial respiration study, early MALSD, especially with obesity, is associated with elevated mitochondrial respiration (40). Since those measurements are maximal respiration rates, which might not reflect actual in vivo flux, you might also make readers aware that your data is consistent with in vivo human studies that found increased hepatic oxidative flux (TCA cycle flux) in obese subjects with moderate steatosis (PMID: 22152305), which appears to wane with severe steatosis and/or inflammation (PMID: 31012869, PMID: 40272888).

Thank you for these suggestions. We have made the suggested changes to the text.

**Reviewer #3 (Recommendations for the authors):**

(1) Throughout the manuscript, the authors refer to the inner mitochondrial membrane, although they never perform assays to distinguish the inner vs outer mitochondrial membrane. It would be better to just refer to the cardiolipin being measured as "mitochondrial."

Thank you. We made these changes.

(2) In figures showing changes in cardiolipins, not all of them change; only a handful of them are reduced in NASH. Could the authors add commentary in the manuscript about what is known about these different cardiolipin species, and speculate as to why certain CLs are changing while others are not?

Thank you. Reviewer #2 had similar comments and we provided additional discussions.

(3) In the human tissues, what do the other mitochondrial inner membrane lipids (PC, PE, PI, PS, LPC, LPE) look like in the healthy vs NASH patients (Figure 1A-D)?

Thank you for this request. We did not include these data in the manuscript as we have a separate ongoing study (the second author is the lead author on this paper) where we are following up on hepatic mitochondrial PS and PE, which we found to be decreased in human MASH samples compared to healthy livers. This turned out to be a convoluted story so we decided not to include it in the paper.

(4) The descriptions of the different MASLD/MASH models are a little sparse. Especially needing more detail is the model for carbon tetrachloride injection, causing NASH. The authors should explain how each of these models typically induces MASLD/MASH.

We now provide these details.

(5) In figures 2E and F, total body mass is unchanged in CLS-LKO mice, but liver mass is decreased; yet on the chow diet, there appears to be lipid accumulation in the liver as well; I am wondering what the authors' reasoning is for this decreased liver mass.

It is difficult to say conclusively, but we suspect it is due to cell death evidenced by fibrosis. It’s important to note that while there is lipid accumulation in the liver, steatosis is relatively mild and the increase in liver triglyceride is quite marginal (Figure 2J).

(6) The lipidomics analysis and comparison of livers in these different models is a wonderful dataset that needs far more depth in terms of unpacking and describing the findings. For example, all the models of MASH show similar changes in most of the lipid species analyzed. NASH appears to be quite different than MASH. This, among other trends, is certainly worth highlighting as it will be of interest to the field.

Thanks for this comment. We agree that while CL phenotype were common to mouse and human MASH samples, there were other changes that we observed in other lipids that may be biologically significant. As described above, we have an ongoing study pursuing mitochondrial PS in the liver.

(7) Figure 2B - It is interesting that the CLS KO only impacts certain CLs. The 72:8 CL, which is regulated by CLS, is also a CL that appears to change in the human patient samples. The information on the specific CL that is changing seems critical to the mechanism of the role of the CL in the disease. Throughout the manuscript, it is important to specify which specific CL is being referred to, instead of broadly characterizing the changes to cardiolipins, especially since most of the cardiolipins shown do not change; only a handful of them do.

Thank you for this suggestion. We have included additional discussions on 72:8 CL in the manuscript.

(8) One potential non-specific mechanism whereby CLS knockout can cause MASH would be if the mice change their overall food consumption. It is an important control to test if the total food intake is different in WT vs KO mice to formally rule out this possibility.

The food intake was not different between the group (Figure 2E).

(9) To determine the extent to which de novo cardiolipin synthesis underlies the change in MASH/fatty liver observed in the HFD, GAN, and CCl4 models in Figure 1, the authors should also put the CLS KO mice on these diets and perform liver histology, analysis of inflammation markers, and analyze immune cell infiltration. Alternatively, the authors could try to rescue the CLS KO model by supplementing cardiolipin in the diet or by injection.

Thank you. We have an ongoing experiment to examine the effect of hepatocyte-specific CLS overexpression on protection from GAN-induced MASLD.

(10) Figure 3F shows a decrease in UQCRC2 by RNA but no change at the protein level in Figure 3H. The authors should comment a bit more on this disparity, and the data in Figure 3F don't mean much for the main point of the study if the levels of the proteins are unchanged.

The reviewer is correct. We initially performed RNAseq in trying to broadly capture how CLS knockout influences liver health, which implicated that transcriptional program for mitochondrial proteins were downregulated. Nevertheless, gold standard measurements of mitochondrial content (mitochondrial protein or mtDNA) did not show change in the abundance with CLS deletion.

(11) The increase in respiration and spare respiratory capacity upon CLS KO shown in Figure 3J is extremely interesting! The explanation of the experiment and its meaning should be significantly expanded upon.

Thank you. We included additional discussion on this point.

(12) Figure 4 - It is interesting that the fraction of the TCA cycle metabolites labeled is increasing with the palmitate tracer and decreasing with the glucose tracer. This implies a "fuel switch," such that more of the TCA cycle carbons originate from fatty acids than glucose upon loss of CLS. The authors should make note of this point. Also, to understand if the total molar quantity of labeling in the TCA cycle from palmitate and glucose is changing, the authors should also report the relative abundance (instead of just the fraction labeled) of the labeled metabolites and unlabeled metabolites.

Thanks for this suggestion, we have now added this discussion.

(13) In Figure 5C-F, the authors show that CLS deletion can activate the caspase pathway, but do not see any change in cytochrome c localization. Can the authors clarify if CLS deletion is sufficient to induce apoptosis?

CLS deletion certainly causes cell death that induces tissue fibrosis. Activation of the caspase pathway suggests that the cell death may be due to apoptosis but we did not see changes in cytochrome c localization. Our lab is currently performing additional experience to test the possibility that CLS deletion may induce ferroptosis.

(14) Figure 6A-C- The authors discuss the I + III2 + IV supercomplex substantially and consistently decreasing in the CLS-KO mice, however, the quantifications do not look statistically significant. Can the authors confirm if these changes are or are not significant and adjust the text accordingly?

The reviewer is correct. Abundances of I+III2+IV supercomplexes are decreased in CLS-LKO mice compared to control mice when quantifying with supercomplex antibody cocktail or with UQCRSF1 (complex III subunit) antibody, but not with complex I antibodies. The discrepancy for these results are not entirely clear but it’s likely a combination of antibody sensitivity and a tricky nature to dissolve high molecular weight protein complexes.

(15) The most compelling data to indicate electron leakage increasing upon CLS knockout is in Figures 7A-E. I would suggest the authors decrease their emphasis on the rearrangement of the supercomplexes and focus their discussion on the very compelling results of Figure 7.

Thanks for this suggestion. We have modified our text.

(16) Figure 7D shows that a major site of electron leak is from site II, and these results also fit with the profound succinate-induced respiration observed in earlier experiments. It would be nice if the authors could test the ability of cardiolipin to rescue these phenotypes, similar to the assay in Figure 5I. Assessing this rescue on the CoQ redox state would also strengthen the claims.

Thank you for this comment. We are encouraged with your suggestions. We have thought about this quite extensively during the preparation of the manuscript but we refrained from making conclusive statements regarding complex II because the magnitude of the increase in electron leak is equally elevated at complex II and III. It’s true that CLS deletion increases succinate-induced respiration, but this might also be because succinate elicits the highest increase in respiration even in wildtype mice (see values in Figure 3K and L compared to other substrates). It would be intriguing to examine the influence of CLS deletion on complex II/III electron leak as well as succinate-induced respiration in tissues where succinate is not a preferred substrate. We have attempted cardiolipin rescue in SUV but unfortunately, we could not get this assay to work for site-specific electron leak measurements.

(17) In Figure 7G-H, it would be nice to see a ratio of oxidized to reduced CoQ, in the CLS deletion mice and in human NASH livers, if samples are available.

Thanks for this suggestion. Data shown (Figure 7- figure supplement 1P-S).

(18) CoQH2 can also deliver electrons to complex II (via its reversal). Complex II shows a remarkable contribution to the electron leak phenotype (Figure 7D). Also, as the complex II monomer showed much larger changes in the native gels of Figure 6 than the complexes involving complex III. A more likely model is that oxidized CoQ accumulates in the CLS knockout model because of increased CoQH2 leak via complex II.

Perhaps. We also thought about this but we are not sure if this fits with the observation that CLS deletion increases succinate-induced respiration, which suggests increased succinate to fumarate conversion, a notion that I am not sure can be congruent with increase CoQH2 reversal to complex II. Overall, I think we lack the tools or evidence to conclusively implicate whether CLS deletion primarily acts on complex II or III. Nevertheless, we appreciate the reviewer’s enthusiasm on these topics as we perform additional experiments on the mechanism of interactions between CL and the ETC.
